# Enzyme catalytic efficiency prediction: employing convolutional neural networks and XGBoost

**DOI:** 10.3389/frai.2024.1446063

**Published:** 2024-10-21

**Authors:** Meshari Alazmi

**Affiliations:** College of Computer Science and Engineering, University of Ha’il, Ha’il, Saudi Arabia

**Keywords:** deep learning, CNN, turnover number, *k*_cat_, enzyme efficiency

## Abstract

**Introduction:**

In the intricate realm of enzymology, the precise quantification of enzyme efficiency, epitomized by the turnover number (*k*_cat_), is a paramount yet elusive objective. Existing methodologies, though sophisticated, often grapple with the inherent stochasticity and multifaceted nature of enzymatic reactions. Thus, there arises a necessity to explore avant-garde computational paradigms.

**Methods:**

In this context, we introduce “enzyme catalytic efficiency prediction (ECEP),” leveraging advanced deep learning techniques to enhance the previous implementation, TurNuP, for predicting the enzyme catalase *k*_cat_. Our approach significantly outperforms prior methodologies, incorporating new features derived from enzyme sequences and chemical reaction dynamics. Through ECEP, we unravel the intricate enzyme-substrate interactions, capturing the nuanced interplay of molecular determinants.

**Results:**

Preliminary assessments, compared against established models like TurNuP and DLKcat, underscore the superior predictive capabilities of ECEP, marking a pivotal shift *in silico* enzymatic turnover number estimation. This study enriches the computational toolkit available to enzymologists and lays the groundwork for future explorations in the burgeoning field of bioinformatics. This paper suggested a multi-feature ensemble deep learning-based approach to predict enzyme kinetic parameters using an ensemble convolution neural network and XGBoost by calculating weighted-average of each feature-based model’s output to outperform traditional machine learning methods. The proposed “ECEP” model significantly outperformed existing methodologies, achieving a mean squared error (MSE) reduction of 0.35 from 0.81 to 0.46 and *R*-squared score from 0.44 to 0.54, thereby demonstrating its superior accuracy and effectiveness in enzyme catalytic efficiency prediction.

**Discussion:**

This improvement underscores the model’s potential to enhance the field of bioinformatics, setting a new benchmark for performance.

## Introduction

The intricate tapestry of cellular metabolism is orchestrated by enzymes and bio-catalysts that expedite and modulate a plethora of biochemical reactions essential for life (Robinson, 2015). Central to understanding an enzyme’s catalytic prowess is the turnover number, *k*_cat_, a metric that quantifies the maximal number of substrate molecules an enzyme can convert to product per active site per unit time (Sánchez et al., 2017; Khodayari and Maranas, 2016). This parameter, emblematic of enzymatic efficiency, serves as a linchpin in the realm of enzymology, underpinning quantitative studies that span from cellular physiology to biotechnological applications (Ebrahim et al., 2016; Davidi et al., 2016). Accurate *k*_cat_ values are indispensable for deciphering the kinetic intricacies of individual enzymes and are foundational in constructing and refining large-scale metabolic models that seek to emulate the holistic metabolic dynamics of organisms (Saa and Nielsen, 2017). Furthermore, with the burgeoning interest in synthetic biology and metabolic engineering, precise knowledge of *k*_cat_ values becomes pivotal in designing enzymes with tailored functionalities (Strutz et al., 2019).

Despite the undeniable significance of *k*_cat_, the landscape of its experimental determination is fraught with challenges. High-throughput experimental assays for *k*_cat_ remain conspicuously absent, rendering acquiring these values for most enzymatic reactions a labor-intensive and costly endeavor (Rives et al., 2021). Consequently, a substantial chasm exists between the number of biochemically characterized enzymes and those with empirically determined *k*_cat_ values (Rao et al., 2019). This paucity of experimental data has spurred the development of computational methodologies aimed at predicting *k*_cat_ values. However, extant prediction frameworks, while pioneering, often grapple with the multifarious and stochastic nature of enzymatic reactions, leading to predictions that, albeit insightful, are occasionally imprecise or bereft of quantified uncertainties (Detlefsen et al., 2022; Smallbone et al., 2013). Traditional methods, relying predominantly on deterministic algorithms, often fall short of capturing the nuanced interplay of molecular determinants governing enzymatic turnover (Goldman et al., 2022).

Artificial intelligence (AI) has significantly advanced in various fields, particularly machine learning (ML) and deep learning techniques. In medicine, deep learning, especially convolutional neural networks (CNN), has revolutionized medical detection systems, especially in analyzing medical images (Salas-Nuñez et al., 2024; Feehan et al., 2021). These developments hold great promise for early diagnosis, detection, and treatment of various diseases, demonstrating the potential of AI to tackle complex real-world problems, particularly in image analysis and computer vision (Du and Swamy, 2014; Pereira and Borysov, 2019). These developments hold great promise for the early diagnosis, detection, and treatment of various diseases, demonstrating the potential of AI to tackle complex real-world problems, particularly enzyme turnover number (*k*_cat_) prediction (Robinson et al., 2020). In this process, CNNs are used to predict the enzymatic turnover by process input data, such as protein and substrate molecular structures. By learning hierarchical representations from input data, CNNs can capture complex patterns and relationships within enzyme-substrate systems, improving prediction performance. Moreover, CNNs provide scalability and efficiency in managing extensive datasets, rendering them invaluable for expediting enzymatic turnover prediction and streamlining enzyme engineering endeavors across diverse biotechnological applications (Upadhyay et al., 2023; Mittal et al., 2021; Ge et al., 2023). The integration of AI into enzyme engineering has led to significant advancements, particularly in tailoring methods for optimizing lipase production and properties such as catalytic activity, stability, and substrate specificity. This progress involves using optimized network models and algorithms to predict and enhance lipase performance. Li et al. (2020) explored various AI-based approaches and their applications in lipase modification, discussing both their benefits and limitations. The author highlights the application of various neural networks and algorithms to optimize lipase production and predict molecular variations affecting its properties. Additionally, they emphasize the need to explore these research gaps and outline future perspectives for AI applications in enzyme engineering, particularly for lipases.

While existing models such as TurNuP and DLKcat have made significant progress in *k*_cat_ prediction, there remains a critical need for methods that predict and quantify the uncertainty inherent in these predictions (Zhou et al., 2020). The biological realm is full of variability and stochasticity, and any model that ignores this inherent uncertainty risks oversimplification and possible misinterpretation (Davidi et al., 2016; Mittal et al., 2021). The enzyme catalytic efficiency prediction (ECEP) seeks to enhance the performance of existing models. It improves predictive accuracy and incorporates a CNNS deep learning algorithm, enabling it to surpass the capabilities of TurNuP and DLKcat. Additionally, we introduce new features derived from enzyme sequences and chemical reaction information, which further enhance performance. By refining the ensemble technique, we achieve significant improvements over current methodologies.

**Figure 1:**
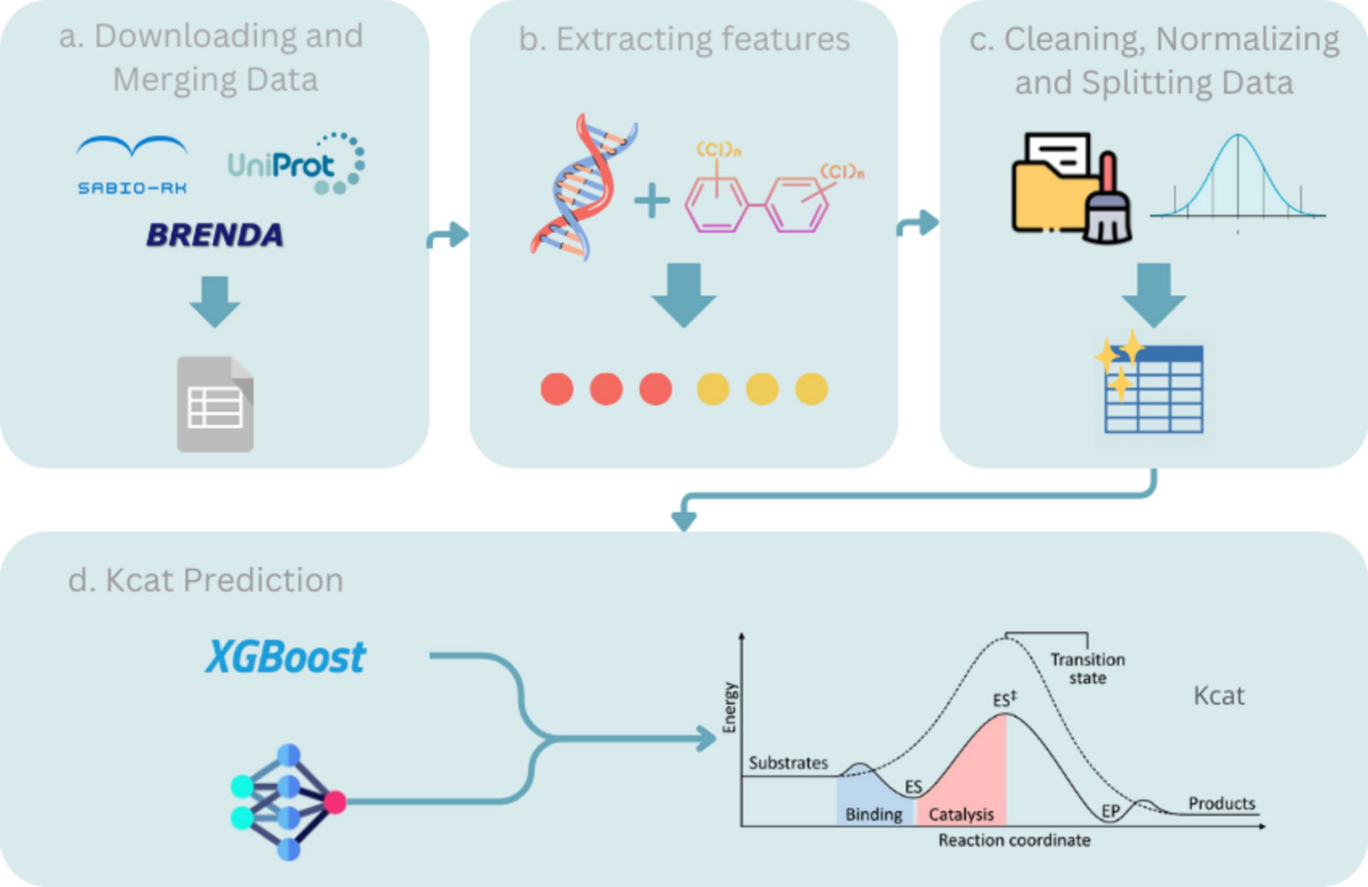
Shows the flowchart and pipeline of the main idea of the method.

While previous implementations such as TurNuP employed simple mean averaging for ensemble predictions, assigning equal weight to each *K*_cat_ prediction, these approaches often failed to account for the varying predictive power of individual models. In this study, we introduce a novel methodology that improves upon this by utilizing a weighted mean approach for ensemble predictions. By optimizing the weights assigned to each model based on their performance, our method ensures that more accurate models have a greater influence on the final prediction. This refinement not only enhances predictive accuracy but also provides a more robust framework for enzyme catalytic efficiency prediction (see Figure 1).

## Background

Enzyme performance prediction using deep learning models has become the cornerstone of contemporary bioinformatics. Kroll et al. (2023) presented a study highlighting TurNuP, a new computational method designed for accurately predicting turnover numbers (*k*_cat_) for enzymatic reactions, which are important for cellular biological activities. Contrary to current models that are limited to specific organisms or are restricted to enzymes closely resembling those in their training data, TurNuP is a generalized model capable of accurately predicting turnover rates for a wide range of enzymes in their natural reactions. TurNuP surpasses previous models and generalizes enzymes different from those in its training set. Incorporation of TurNuP-*k*_cat_ values into metabolic models heightens predictions of proteome allocation. Li et al. (2022) highlighted DLKcat, a deep learning model that helps in the prediction of the turnover number (*k*_cat_) of enzymes. Unlike conventional methods, DLKcat demands less elaborate input characteristics, focusing mainly on information about the amino acid sequence of the enzyme and one of the reaction substrates. The model is intended to deliver accurate predictions of *k*_cat_ especially when experimental data for closely related enzymes are lacking. DLKcat is imagined to have wide application in various enzymatic reactions. However, its efficiency drops significantly for enzymes that differ from those in the training dataset. This shortcoming highlights the significance of training deep learning models on dissimilar and representative datasets to guarantee vigorous performance across a broad range of enzyme types. Nevertheless, DLKcat is assumed as a tool to predict *k*_cat_ values, especially in scenarios where experimental data for the respective enzymes are lacking.

Heckmann et al. (2020) demonstrated the potency of machine learning in the estimation of catalytic turnover numbers (*k*_cat_) of enzymes in *Escherichia coli* (*E. coli*). In particular, the study discovered novel protein structures corresponding to catalytic turnover, enlightening previously unexplored aspects of enzyme kinetics. The forecasting models developed in this research have broader implications. These models surpass previous methods, resulting in significantly better accuracy in predicting quantitative proteome data. They provide a powerful tool for investigating the complexities of cellular metabolism, helping to elucidate growth rates, proteome composition, and organismal physiology. Consequently, these developments offer a valuable tool for comprehensively understanding metabolism and the proteome at the genome-scale. Wendering et al. (2023) analyzed that turnover numbers are the main descriptors of enzyme activity. Integrating them into constraint-based metabolic modeling holds the promise of improving the prediction accuracy of various cellular properties. Despite efforts to integrate *in vitro* and *in vivo* turnover data, current methods provide inadequate predictions of condition-dependent growth rates in *E. coli* and *Saccharomyces cerevisiae* (*S. cerevisiae*), especially when considering protein abundance. The authors propose a new method that combines proteomic and physiological data to estimate turnover rates, leading to better predictions of growth rates under specific conditions. This approach not only increases accuracy but also offers a way to catalog the catalytic efficiency of other organisms, thereby advancing our understanding of cellular metabolism.

Tachibana et al. (2023) applied the Bayesian algorithm to enhance enzyme-catalyzed reactions, rooted in the probabilistic interpretation of knowledge, it has witnessed a renaissance in the field of bioinformatics and computational biology. Unlike frequentist approaches, which offer static point estimates, Bayesian methodologies provide a probabilistic framework, allowing for the incorporation of prior knowledge and the quantification of uncertainty in predictions (Shields et al., 2021; Braconi, 2023). This is particularly salient in enzymology, where data scarcity often impedes robust predictions. By leveraging prior distributions and updating them with new data through Bayes’ theorem, Bayesian methods offer a dynamic and adaptive approach to knowledge synthesis (Wang et al., 2023).

The ECEP methodology utilizes convolutional neural networks (CNNs), a sophisticated deep learning algorithm, to predict enzymatic turnover rates. CNNs are adept at autonomously extracting and learning complex feature patterns from intricate datasets (Memon et al., 2020). In this context, the input data consists of enzyme sequences and chemical reaction information. Through the analysis of these inputs, CNNs discern hidden features and patterns essential for comprehending enzymatic activities. These extracted features empower the model to precisely predict enzymatic turnover values, offering significant insights for biochemical research and applications (Gao et al., 2019). This approach harnesses the capabilities of CNNs in managing high-dimensional biological data, establishing it as a potent tool for enzymatic prediction tasks (Sikander et al., 2021).

## Materials and methods

We incorporated TurNuP, which involves preprocessing data for model training, including the generation of datasets through the utilization of chemical reaction information.

### Data sources

In our research, we employed the *k*_cat_ dataset synthesized from the integration of multiple databases, thereby facilitating the development of advanced machine-learning models aimed at predicting enzyme turnover numbers. This dataset was deposited in Zenodo, ensuring public accessibility to uphold the principles of transparency and reproducibility inherent in our findings. The primary dataset amalgamated for this study underwent meticulous curation, drawing from three bioinformatic repositories.

- BRENDA: Renowned as a comprehensive enzyme information system, BRENDA furnishes intricate enzyme and metabolic data meticulously extracted from the primary literature. Its indispensability lies in the facilitation of biochemical pathway reconstruction and enzyme characterization (Schomburg et al., 2017).
- UniProt: A global repository of protein sequences and functional data. UniProt serves as a pivotal resource for associating protein sequences with functional insights, thus proving instrumental in elucidating the biochemical roles of the enzymes (The UniProt Consortium, 2018).
- Sabio-RK: Specialized repositories focusing on enzyme kinetics. Sabio-RK offers curated kinetic data about enzyme-catalyzed reactions, providing critical parameters, such as reaction rates and environmental conditions (Wittig et al., 2018).

The collaborative integration of these databases bolsters the robustness of our dataset, thereby empowering precise predictions of enzymatic activity through the application of sophisticated machine-learning methodologies. For comprehensive elucidation and access to the dataset, we refer to its deposition on Zenodo and its associated publication in Nature Communications.

This will give us a training set with 3,391 entries, and a testing set with 874 entries.

### Preprocessing steps

We adopted an approach similar to that outlined in the TurNuP paper, with the addition of generating new features derived from the substrate and product components of chemical reactions.

- Data integration: Information sourced from the aforementioned repositories was amalgamated based on enzyme commission (EC) numbers and protein identifiers, thereby facilitating seamless fusion of enzyme sequences with their corresponding *k*_cat_ values.
- Feature extraction: Pertinent attributes for each enzyme were abstracted, encompassing the conversion of amino acid sequences into numerical representation by utilizing the pre-trained ESM1b model and its fine-tuned version. Additionally, binary numerical representations of chemical reactions were computed using three methodologies: structured fingerprinting, difference fingerprinting, and the DRFP.
- Data cleaning: Instances with missing, duplicate, or outlier values were removed to ensure data integrity.
- Normalization: Numerical features were normalized to ensure uniform scaling by employing the *Z*-score normalization technique.
- Train/test splitting: The dataset was partitioned into a training set, utilized for model training, and a test set, employed for unbiased evaluation of the model performance post-training. This approach ensures the model’s ability to generalize effectively to new, unseen data, thereby upholding the reliability and robustness of the predictive models.

### Final useful features

Upon the conclusion of preprocessing, we acquired the final train and test pickles, highlighting notable features embedded within this dataset.

A critical component of the ECEP model’s success is the detailed feature engineering process. Below, we outline the steps taken to extract and process features from raw data:

1. Data integration: We amalgamated enzyme sequence data with their corresponding *k*_cat_ values from BRENDA, UniProt, and Sabio-RK databases.
2. Sequence representation: Enzyme sequences were converted into numerical vectors using the ESM-1b, and ESM_1b_ts models which provided 1,280-dimensional feature vectors for each sequence.
3. Reaction fingerprinting: Chemical reactions were represented using three fingerprinting methodologies:

o Structural fingerprinting: Captured physical and chemical properties (length: 4,096 binary values).
o Differential fingerprinting: Encoded differences between reactants and products (length: 2,048 binary values).
o Difference fingerprinting: Quantified alterations in chemical reactions (length: 2,048 binary values) (see Table 1).

**Table 1:** List of features used in ECEP.

Feature name	Description
ESM_1b model representation	Numerical vector representation of enzyme sequences
ESM_1b_ts model representation	Numerical vector representation of enzyme sequences
ESM combined (used only for CNN)	ESM_1b vector multiplied by ESM_1b_ts vector
Structural fingerprint	Binary representation of structural attributes
Differential fingerprint	Binary encoding of reactant-product differences
Difference fingerprint	Quantification of reaction alterations

### Enzyme sequence representation

Enzyme sequences, initially presented as extensive strings of capitalized characters in fasta file format, necessitate conversion into a numerical format conducive to machine learning models. Leveraging the ESM-1b model, a transformer-based protein language model trained through unsupervised methods on vast protein sequences, these sequences are transformed into numeric vectors of length 1,280. The initial iteration of ESM-1b yields a standard numeric representation, while its fine-tuned counterpart, ESM-1b_ts, furnishes an adjusted numeric vector tailored to specific sequence intricacies. Both representations serve as instrumental components for the deep learning models deployed in the study, facilitating the effective prediction of enzyme characteristics.

### Reaction fingerprinting

Utilizing the same approach employed for reaction fingerprinting in chemical reaction representation, the process entails converting reactions into numerical representations. Three distinct fingerprinting methodologies are elucidated: structural fingerprinting, differential fingerprinting, and difference fingerprinting. Each methodology adopts a unique perspective on analyzing and representing chemical reactions in numeric or symbolic formats to facilitate diverse computational tasks.

- Structural fingerprinting: This methodology primarily concentrates on capturing the structural attributes of molecules engaged in reactions. It typically entails encoding the physical and chemical properties of each molecule, culminating in a detailed representation conducive to tasks such as similarity assessment and machine learning modeling. The structural fingerprint spans a length of 4,096, comprising binary values of 0 or 1.
- Differential fingerprinting: Differential fingerprinting extends beyond individual characteristics of reactants and products by explicitly encoding disparities between them. This approach proves particularly advantageous in predictive modeling scenarios, where the objective is to discern how alterations in reactant structures manifest in product configurations. The differential fingerprint spans a length of 2,048, featuring binary values of 0 or 1.
- Difference fingerprinting: Analogous to differential fingerprinting, this methodology focuses on delineating alterations occurring throughout a reaction. However, it often employs more complex algorithms to dissect and quantify the precise nature of these alterations, thereby providing deeper insights into reaction mechanics. This encompasses transformations of chemical groups, bond formation or cleavage, and other significant changes during the reaction process. The difference fingerprint spans a length of 2,048.

### *k*_cat_ predictions

Our predictive models for *k*_cat_ values encompass both traditional machine learning and deep learning approaches.

### XGBoost

XGBoost, a prominent algorithm in our research arsenal, is celebrated for its exceptional precision in handling regression tasks, including *k*_cat_ value prediction. Operating within a gradient-boosting framework akin to its implementation in TurNuP, XGBoost constructs decision trees sequentially, iteratively refining predictions by addressing prior errors.

**Figure 2:**
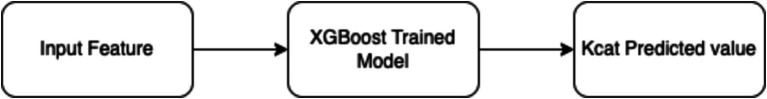
XGBoost trained model predicts *k*_cat_ value.

This algorithm incorporates regularization techniques to mitigate overfitting, employs tree pruning to enhance model simplicity, and leverages parallel processing for expedited computation. Moreover, its intrinsic support for cross-validation and adaptable loss functions enables tailored model optimization, rendering it highly adept at accommodating the intricacies of our expansive biochemical datasets (see Figure 2).

### Convolution neural network

**Figure 3:**
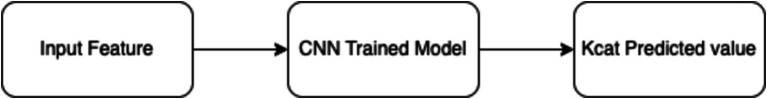
CNN trained model predicts *k*_cat_ value.

Convolutional neural networks (CNNs) represent a class of deep learning algorithms traditionally employed for image recognition and classification tasks. However, their adaptability extends to regression problems, including the prediction of enzyme kinetics such as *k*_cat_ values. In our study, CNNs are harnessed to glean patterns and features from input data, comprising structural or sequence data about enzymes alongside numerical representations of chemical reactions (see Figure 3).

CNN architectures are characterized by multiple layers, including convolutional layers, pooling layers, and fully connected layers. Initially, the train and test datasets are loaded, essential features are filtered, and these features are vertically concatenated to form a multidimensional array, sequentially traversing each layer of the CNN. Convolutional layers apply filters to the input data, capturing local patterns and features, while pooling layers serve to reduce the spatial dimensions of the resultant feature maps, thereby mitigating computational complexity and curbing overfitting. Subsequently, fully connected layers amalgamate the features gleaned from preceding layers to facilitate predictions.

Training a CNN entails furnishing it with labeled data—comprising enzyme sequences, numerical representations of chemical reactions, and their corresponding *k*_cat_ values—and optimizing its parameters, namely weights and biases, via methodologies such as backpropagation and gradient descent.

**Figure 4:**
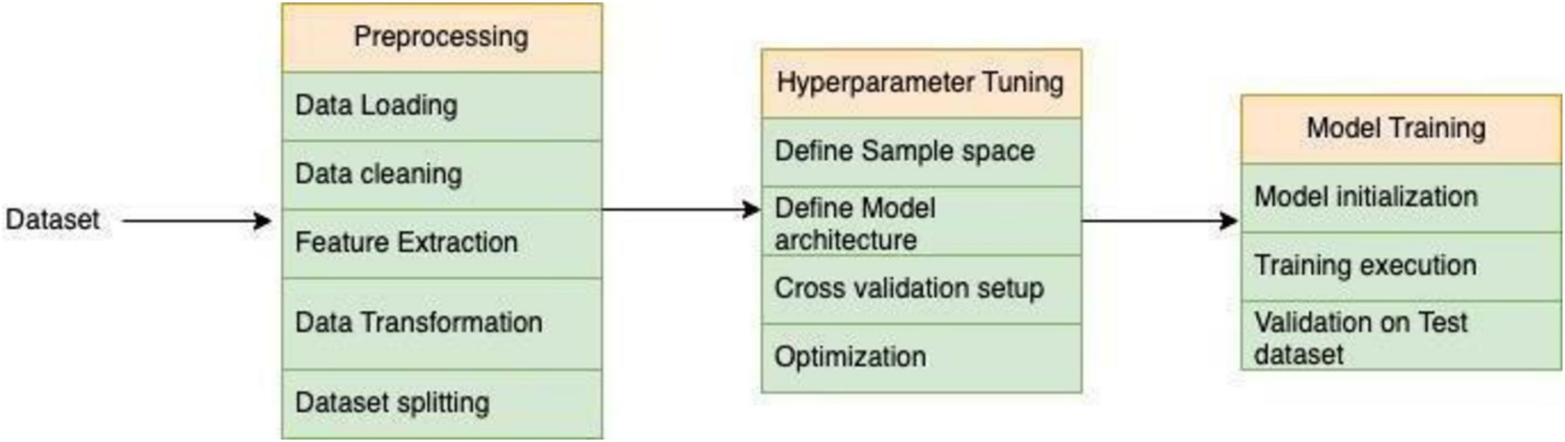
Loading the dataset for preprocessing, getting the optimized hyperparameter, used that hyperparameter to train the Model.

By leveraging CNNs for *k*_cat_ value prediction, we capitalize on their innate capacity to autonomously extract pertinent features from input data, potentially encapsulating intricate relationships between enzyme structures or sequences and their catalytic efficacy. This affords the prospect of achieving more precise predictions vis-à-vis traditional regression techniques, particularly when confronted with high-dimensional or nonlinear datasets (see Figure 4).

### Model evaluation and validation

In order to gauge the efficacy and resilience of both the convolutional neural network (CNN) and XGBoost models, stringent evaluation protocols were implemented. The dataset underwent partitioning into distinct training, validation, and test sets, ensuring exposure of the model to a diverse array of enzymatic reactions throughout both the training and evaluation phases. For XGBoost, Cross-validation, a methodology involving further division of the training set into multiple folds, was employed to furnish a more intricate assessment of the model’s performance. This iterative procedure, wherein various folds are designated as validation sets in each iteration, provides an exhaustive perspective on the model’s capacity for generalization.

### Hyperparameter tuning

The process of selecting optimal hyperparameters is paramount, as it intricately refines the configuration of the trained model, ensuring optimal performance across diverse datasets and tasks. Through systematic adjustment of these parameters, we augment the model’s capacity to discern intricate patterns and correlations within the data, thereby enhancing predictive accuracy and generalization capability. This iterative procedure entails experimenting with various hyperparameter combinations and assessing their impact on the model’s performance, with the aim of striking a delicate balance between model complexity and predictive prowess. The chosen hyperparameters wield significant influence over the model’s behavior and efficacy, underscoring the critical nature of their selection within the machine learning pipeline.

### XGBoost hyperparameter tuning

In our XGBoost implementation, we harnessed the capabilities of the hyperOpt package in Python to streamline the selection of optimal hyperparameters. This sophisticated tool automates the intricate process of hyperparameter tuning, allowing us to systematically explore the hyperparameter space and pinpoint configurations that yield superior model performance. Building upon the methodology detailed in the previous implementation elucidated in the TurNuP paper, we conducted a rigorous search for the most appropriate combination of hyperparameters.

This endeavor entailed defining a comprehensive search space encompassing a multitude of hyperparameters, including but not limited to learning rate, tree depth, and regularization terms. Through iterative evaluation of diverse parameter configurations and meticulous assessment of their impact on model performance, our objective was to identify settings that optimize predictive accuracy and generalization capability. This meticulous approach ensures that our XGBoost model is finely attuned to the nuances of our dataset, thereby augmenting its efficacy in predicting enzyme *k*_cat_ values. Table 2 indicates the detail of the hyperparameter tuning search space.

**Table 2:** Hyperparameter search spaces for XGBoost algorithm.

Hyperparameter	Search spaces
Learning rate	Adjusts the step size during each iteration while progressing towards minimizing a loss function. Range: 0.01 to 1
Max depth	Determines the maximum depth of the trees, thus regulating overfitting. Range: 4 to 12
Reg lambda	Utilizes L2 regularization on weights to control overfitting by penalizing complex models. Range: 0 to 5
Max delta step	Sets the maximum delta step allowed for updating the weight estimation of each tree. Range: 0 to 5
Min child weight	Specifies the minimum sum of instance weight (hessian) required in a child to counteract overfitting. Range: 0.1 to 15
Number of rounds	Denotes the quantity of training rounds or trees to construct, influencing the complexity of the model. Range: 20 to 200

### Convolutional neural network hyperparameter tuning

In our pursuit of optimal CNN performance, we embraced a randomized approach within the defined search space for hyperparameter selection. This expansive search space encapsulated a spectrum of parameters, including the number of filters, kernel dimensions, neurons in the fully connected layer, choice of optimizer, batch size, and dropout rate. Furthermore, we incorporated callback functions to dynamically adjust the learning rate and establish early stopping criteria, thus mitigating concerns related to overfitting. Acknowledging the intrinsic variability in deep learning outcomes stemming from random weight initialization and data shuffling, we implemented an ensemble learning strategy to bolster model robustness.

Specifically, we amalgamated the predictions from three distinct models utilizing a weighted averaging technique. This methodology allocates greater weights to the predictions of well-performing models, thereby magnifying their impact within the final prediction ensemble. Through the utilization of this ensemble approach, our aim was to alleviate the inherent variability in deep learning models and bolster the reliability of our predictions. Table 3 indicates the detail of the hyperparameter tuning search space.

**Table 3:** Hyperparameter search spaces for CNN.

Hyperparameter	Sample spaces
Number of filters in convolutional layers	The quantity of filters in convolutional layers escalates progressively across layers to effectively capture intricate features. For the initial layer, the filter count ranges from 2 to 15, followed by 4 to 25 for the second layer, and 8 to 35 for the third layer, each incremented by 2
Kernel size in convolution layers	Kernel size plays a pivotal role in determining the receptive field size, crucial for feature extraction. In the initial layer, the kernel size varies from 3 to 19, followed by 5 to 17 for the second layer, and 7 to 15 for the third layer, with increments of 2, ensuring odd numbers are utilized
Neurons in fully connected layers	The quantity of filters in convolutional layers escalates progressively across layers to effectively capture intricate features. For the initial layer, the filter count ranges from 2 to 15, followed by 4 to 25 for the second layer, and 8 to 35 for the third layer, each incremented by 2
Optimizers	Optimizers play a crucial role in modulating learning speed and minimizing loss. Available options encompass Nadam, Adam, and RMSprop, each offering distinct advantages in optimizing model performance
Batch sizes	The selection of batch sizes profoundly impacts training stability and speed. Options span from 8 to 128, allowing for flexible adjustments to accommodate varying computational resources and training requirements

## Results

The fruition of any research undertaking, particularly within the realm of computational biology and machine learning, culminates in the presentation of results. Within the scope of the “ECEP” model, crafted to forecast enzymatic turnover numbers, the results serve as more than mere evidence of the model’s effectiveness; they symbolize its potential to catalyze advancements within the broader scientific community.

The advent of the “ECEP” model ushers in a new era of enzymatic turnover prediction. Our implementation techniques, encompassing both XGBoost and the newly introduced CNN deep learning approach, have surpassed the performance benchmarks set by previous methodologies.

### XGBoost

Through enhancements to the previous XGBoost results, we have attained the following updated outcomes. Employing an ensemble technique, we amalgamated the results from all trained models using a weighted average mean. This comprehensive approach resulted in refined predictions, further elevating the model’s performance and predictive accuracy.

**Figure 5:**
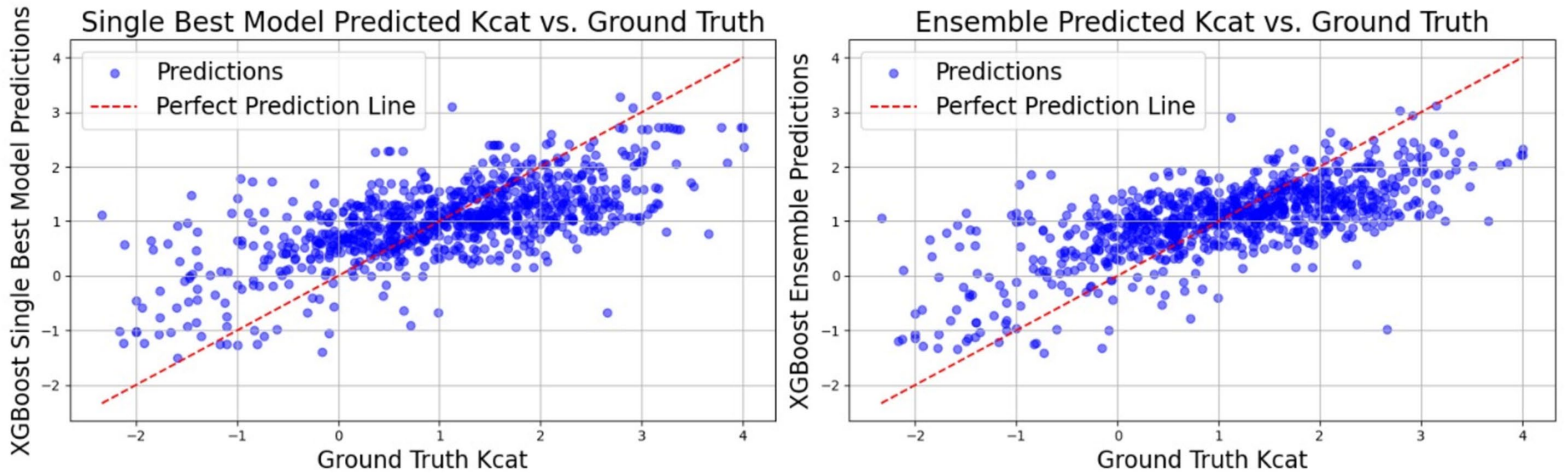
The graph illustrates the relationship between predicted *k*_cat_ values (obtained by training XGBoost using previous features) and ground truth *k*_cat_ values. The red line represents the ground truth predictions, while the blue dots signify predictions generated by the individual XGBoost-trained models. Notably, the predictions from the XGBoost Ensemble model appear to cluster closely around the ground truth predictions, indicating a higher level of precision and agreement between the predicted and actual values compared to the predictions from the single XGBoost model.

Table 4 indicates XGBoost results were notably enhanced with only previous features within a single model, as well as by ensembling the predictions from the best-performing models. This dual strategy yielded a significant improvement in predictive performance, underscoring the efficacy of feature augmentation and ensemble methodologies in refining model outcomes (see Figure 5).

**Table 4:** XGBoost results with single best model and ensemble by utilizing the new features.

Metrices	Single best model	Ensemble
*R*^2^ Score	0.43	0.47
Mean square error	0.77	0.71
Pearson coefficient	0.66	0.68

### Convolution neural network

**Figure 6:**
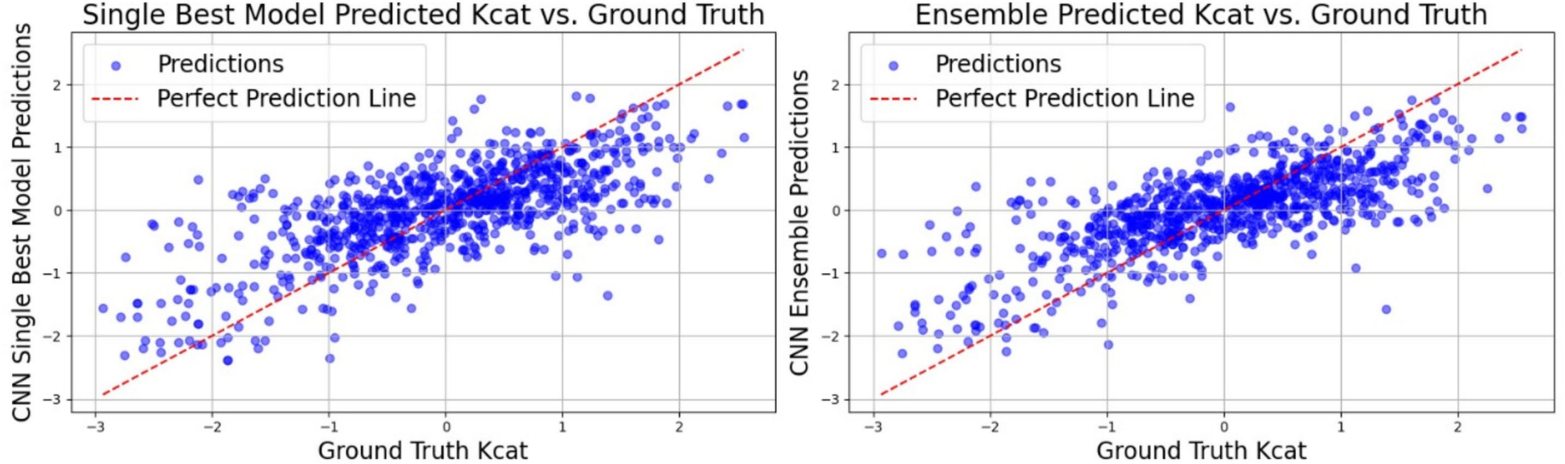
In the graph depicting the relationship between predicted *k*_cat_ values (obtained through CNN models trained on previous feature only) and ground truth *k*_cat_ values, the red line represents the ground truth predictions, while the blue dots signify predictions generated by the individual CNN-trained models. Notably, the predictions from the CNN Ensemble model appear to cluster closely around the ground truth predictions, indicating a higher level of precision and agreement between the predicted and actual values compared to the predictions from the single CNN model.

Convolutional neural networks (CNNs) have markedly enhanced the predictive performance of our model. Not only we have optimized the performance of existing features from the TurNuP methodology, but we have also elevated model efficacy by incorporating new features derived from enzyme sequences and chemical reactions. The ensuing tables delineate the outcomes achieved with and without the integration of these new features (see Figure 6 and Table 5).

**Table 5:** This table illustrates the CNN results attained through the utilization of legacy features within a single model, as well as via the aggregation of predictions from the most proficient models in an ensemble.

Metrices	Single best model	Ensemble
*R*^2^ Score	0.49	0.54
Mean square error	0.51	0.46
Pearson coefficient	0.70	0.73

This dual approach has yielded notable enhancements in predictive accuracy and model performance, underscoring the effectiveness of leveraging both individual model predictions and ensemble methodologies in refining overall outcomes.

### Ensembling

In contrast to the previous TurNuP implementation, where ensemble averaging was conducted by simply taking the mean of all model predictions which gives equal weightage to each predicted value of *k*_cat_, we have enhanced our ensemble methodology by employing a weighted mean approach. This refined method optimizes weights for each model’s predictions by minimizing a loss function against true values. It ensures that weights sum to one and are constrained between zero and one. By dynamically adjusting weights based on predictive performance, more accurate models exert a greater influence on the final prediction, thereby enhancing the ensemble’s robustness and accuracy compared to simpler averaging methods.

While it is possible to ensemble predictions from all trained models, doing so would escalate computational costs. Thus, by ensembling only the best-performing model and leveraging diverse learning features from this model, we can mitigate computational expenses while still achieving superior ensemble performance.

### Comparative analysis

To truly appreciate the prowess of the “ECEP” model, it’s essential to juxtapose its performance against that of its contemporaries. When benchmarked against models like DLKCat and TurNuP, “ECEP” consistently outperformed in terms of both *R*^2^ and mean squared error (MSE). Such comparative superiority is not just a testament to “ECEP”’s advanced architecture but also its adaptability to the nuances of enzymatic data. A deeper dive into the results reveals some intriguing insights.

The landscape of enzymatic turnover prediction has been punctuated by several innovative models over the years. However, the introduction of “ECEP” has stirred the waters, prompting a re-evaluation of established methodologies. To truly gauge the prowess of “ECEP,” it’s imperative to benchmark it against its contemporaries, notably TurNuP and DLKcat. Before embarking on the comparative analysis, it’s imperative to delve into the historical context. Both TurNuP and DLKcat have established themselves as stalwarts in the field, each offering a distinctive approach:

- TurNuP: Rooted in deep learning architectures, TurNuP harnessed the formidable capabilities of neural networks to unravel the intricate relationships within enzymatic data. Its multi-layered design was crafted to capture both linear and non-linear patterns, rendering it a preferred choice among researchers for numerous years.
- DLKcat: In contrast, DLKcat employed a hybrid approach, amalgamating traditional regression techniques with machine learning algorithms. Its hallmark was its adaptability, with the model being meticulously tailored to accommodate various enzyme families and experimental conditions.

### Performance metrics a side-by-side view

#### *R*-squared (coefficient of determination)

**Figure 7:**
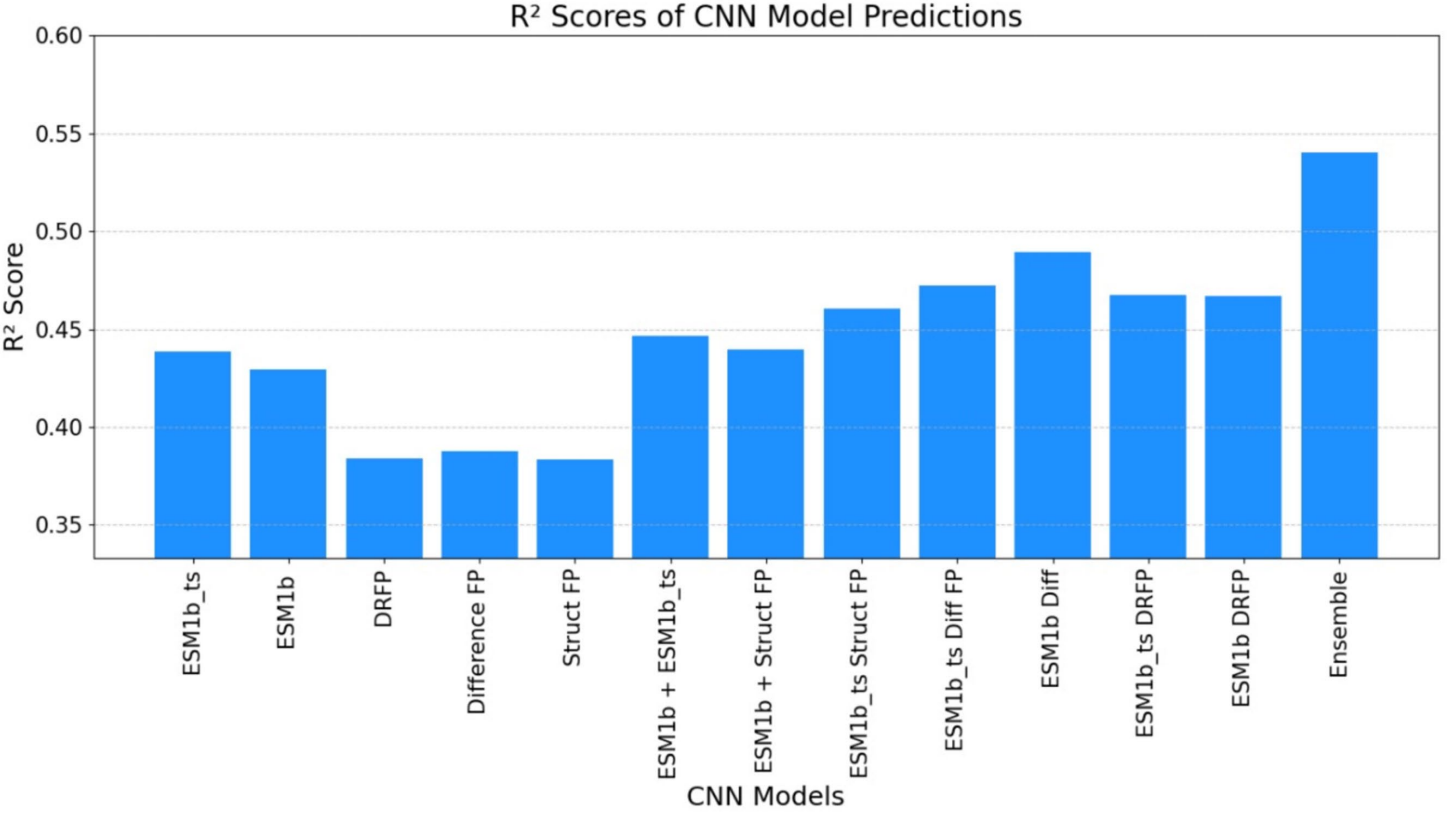
The graph below illustrates the *R*-squared (*R*^2^) score obtained by the ECEP model, which was trained using the CNN approach. This depiction showcases the model’s performance in capturing the variance in the observed *k*_cat_ values, thereby providing insights into its predictive capabilities.

**Figure 8:**
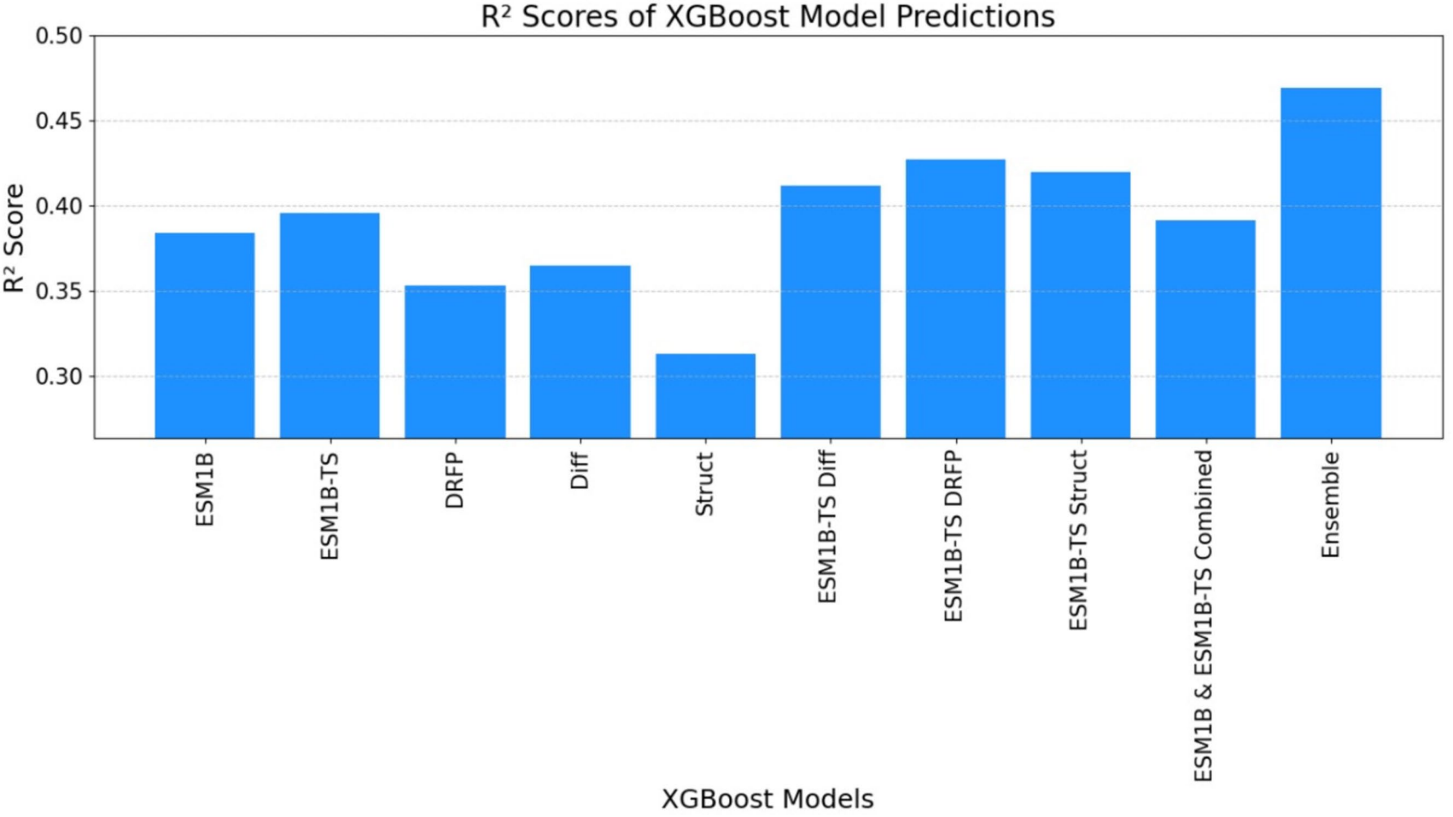
The graph illustrates the *R*-squared (*R*^2^) score obtained by the ECEP model, trained using the XGBoost.

- ECEP: With an *R*^2^ value of 0.54, “ECEP” showcased its superior predictive capabilities, capturing 55% of the variance in the observed *k*_cat_ values.
- TurNuP: TurNuP, with its deep learning foundation, achieved an *R*^2^ value of 0.44. While commendable, it fell short of the benchmark set by “ECEP.”
- DLKcat: DLKcat’s hybrid approach yielded an *R*^2^ of 0.44, reflecting its balanced methodology but also highlighting areas of potential improvement (see Figures 7, 8).

#### Mean squared error

**Figure 9:**
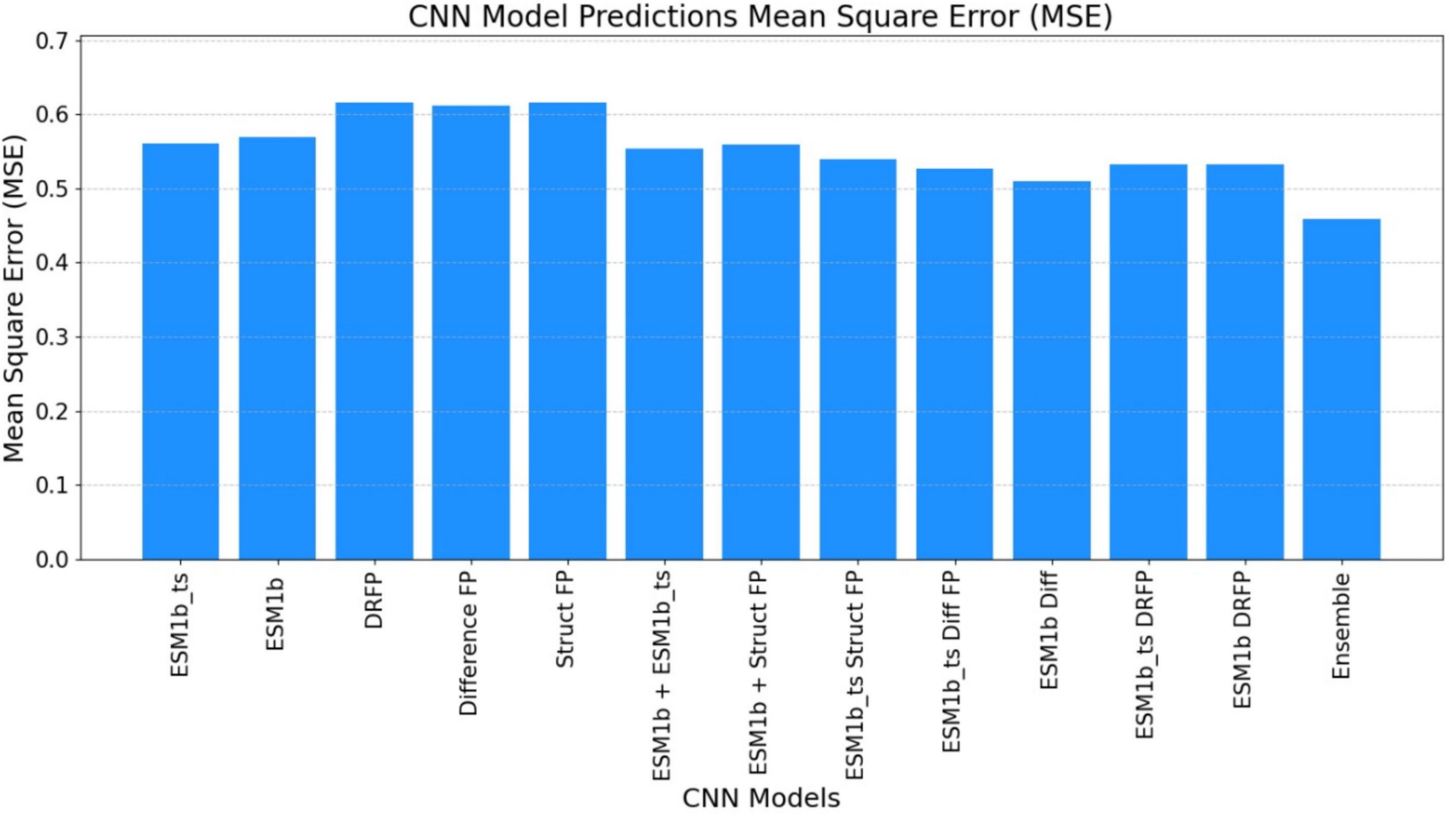
The mean square error (MSE) obtained by the ECEP model, trained using the convolutional neural network (CNN), was 0.46, underscoring the model’s precision and reliability.

**Figure 10:**
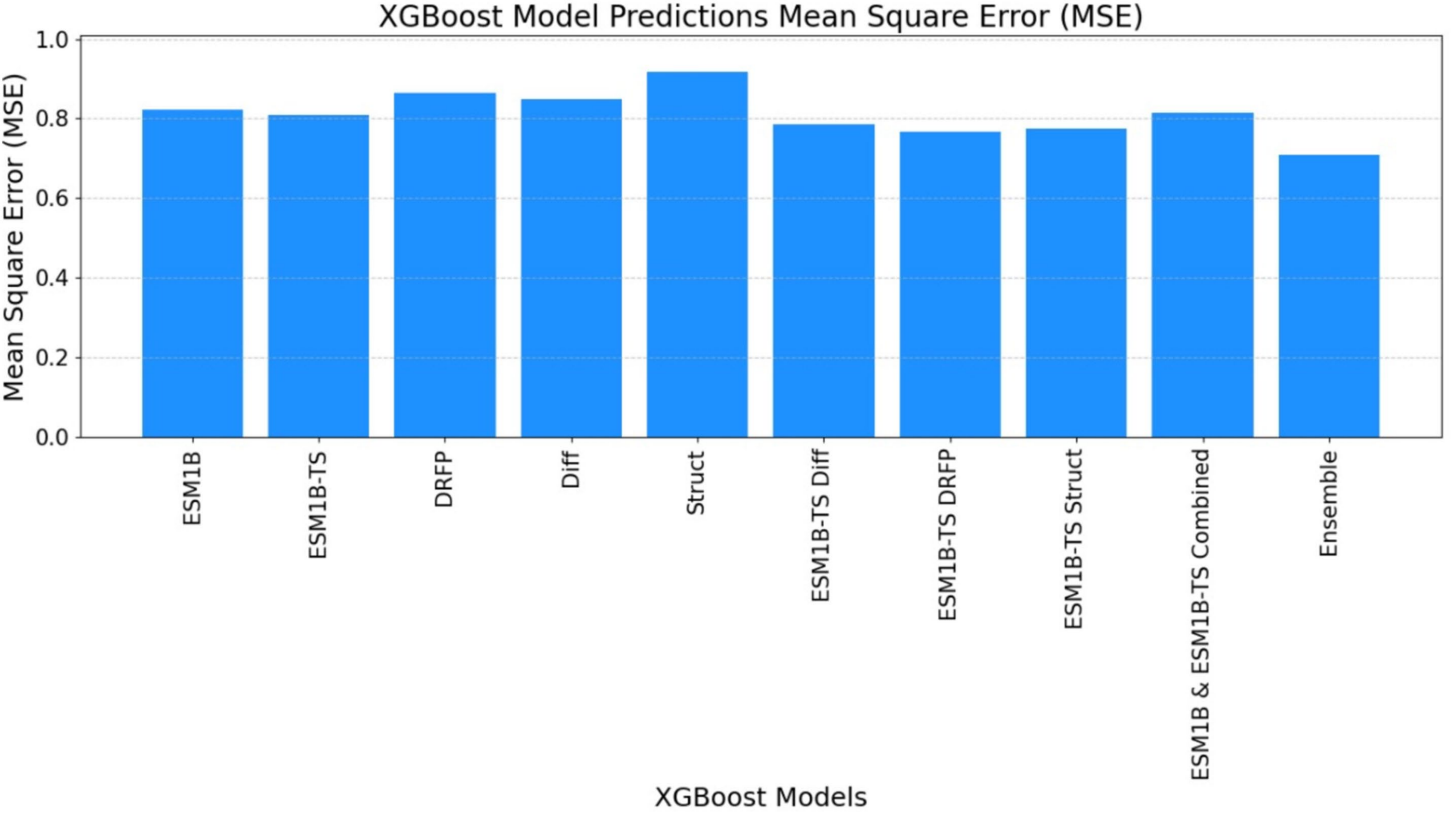
The mean square error (MSE) obtained by the ECEP model, trained using the XGBoost algorithm, was 0.69, affirming the model’s precision and reliability.

**Figure 11:**
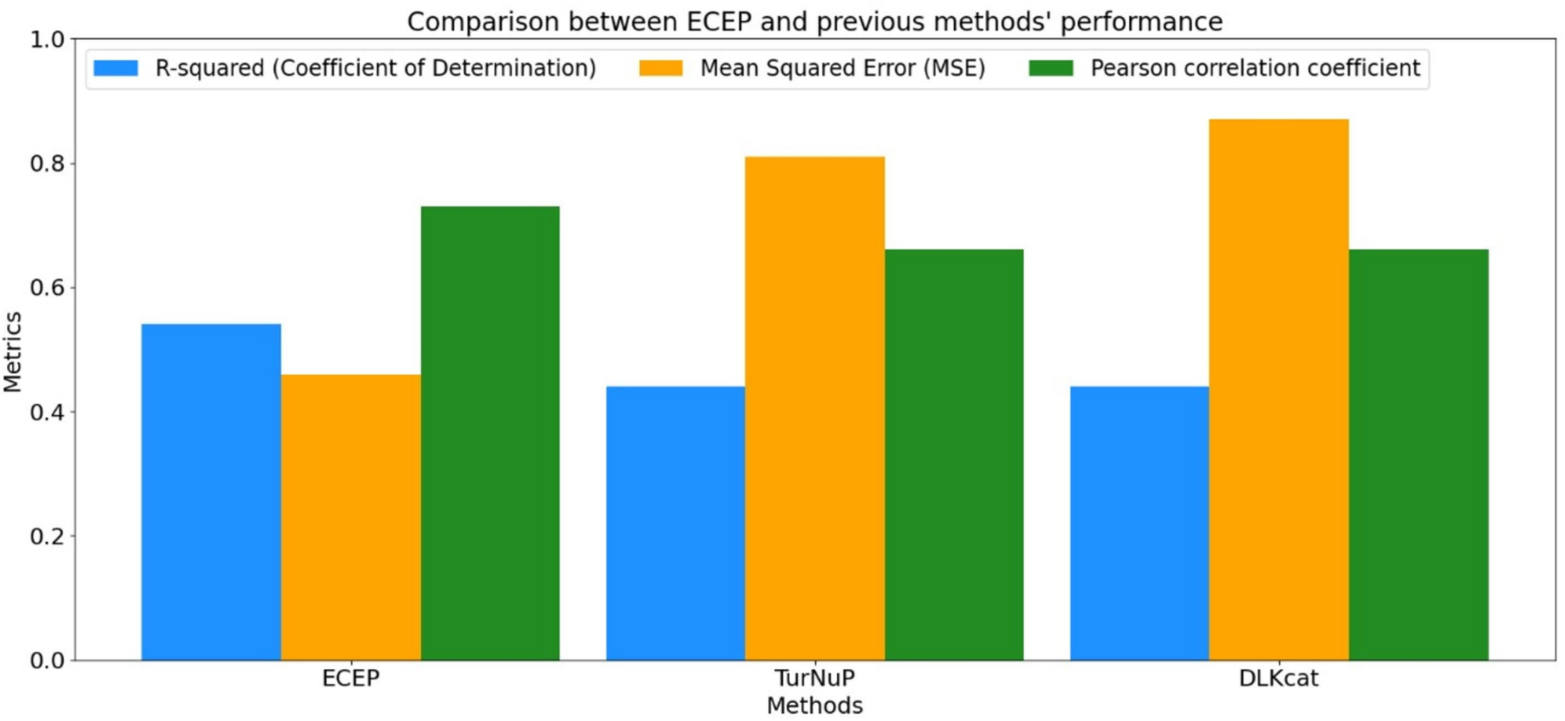
Comparison between *R*-squared, MSE, and Pearson correlation coefficient obtained by ECEP and previous methods; TurNuP, and DLKcat.

- ECEP: “ECEP” demonstrated remarkable precision and reliability with an MSE of 0.46, indicative of its superior performance.
- TurNuP: TurNuP achieved an MSE of 0.81, showcasing impressive performance but also highlighting areas for potential refinement to enhance accuracy.
- DLKcat: DLKcat recorded an MSE of 0.87, reflecting the inherent challenges associated with its hybrid approach and the need for careful balancing of traditional and machine learning techniques (see Figures 9–11 and Table 6).

**Table 6:** This table illustrates a comparison of evaluation metrics scores across various techniques.

Evaluation metrics	ECEP score	TurNuP score	DLKcat score
*R*-squared (coefficient of determination)	0.54	0.44	0.44
Mean squared error (MSE)	0.46	0.81	0.87
Pearson correlation coefficient	0.73	0.66	0.66

### Underlying architectures: strengths and limitations

- ECEP: ECEP represents a significant advancement over previous implementations such as TurNuP, leveraging the convolutional neural network algorithm to elevate the *R*^2^ score from 44% to an impressive 55%. Its innovative approach not only enhances predictive accuracy but also provides a transparent and interpretable framework.
- TurNuP: TurNuP’s deep learning architecture empowers it to effectively model complex, non-linear relationships within enzymatic data. Its multi-layered neural network excels at capturing intricate patterns, making it highly adaptable to diverse datasets. However, TurNuP’s reliance on extensive data for training and its inherent black-box nature can pose challenges in terms of interpretability, limiting its utility in certain applications.
- DLKcat: DLKcat’s hybrid approach combines traditional regression techniques with machine learning algorithms, endowing it with the flexibility to adapt to various data types and enzyme families. While commendable, this versatility sometimes leads to challenges in finding the optimal balance between regression and machine learning methodologies. DLKcat’s performance highlights its potential for refinement and optimization to achieve even greater predictive accuracy.

The introduction of ECEP has set new standards in enzymatic turnover prediction. While TurNuP and DLKcat have contributed significantly to the field, ECEP emerges as a frontrunner due to its blend of accuracy, flexibility, and interpretability. Nonetheless, scientific progress thrives on collaboration and the integration of insights from diverse methodologies. By amalgamating insights from ECEP, TurNuP, and DLKcat, future methodologies can advance even further, paving the way for more sophisticated approaches in enzymatic turnover prediction.

One of the challenges posed by “ECEP” is its computational intensity, especially with large datasets. The complexity of processing and analyzing vast amounts of data can lead to significant computational demands, which may limit the practical application of the model in real-world scenarios. Future research can focus on optimizing the model for scalability, ensuring that it remains efficient even as the data volume grows. This could involve developing more advanced algorithms that reduce computational load, enhancing parallel processing capabilities, or leveraging high-performance computing resources to manage larger datasets more effectively.

As the field of enzymology evolves, new types of data, from molecular dynamics simulations to quantum mechanical calculations, emerge. These advancements offer detailed insights into enzyme behavior and interactions at unprecedented levels of precision. Adapting “ECEP” to incorporate and leverage these data types can enhance its predictive capabilities, allowing it to provide more accurate and comprehensive analyses. This adaptation could involve integrating new computational techniques and data sources into the model, as well as refining its algorithms to handle the increased complexity and volume of information.

Furthermore, collaboration with other scientific disciplines, such as bioinformatics and structural biology, may provide additional insights and methodologies that can be incorporated into “ECEP.” Such interdisciplinary approaches could lead to the development of hybrid models that combine the strengths of various computational and experimental techniques, thereby improving the overall performance and applicability of “ECEP.”

In summary, addressing the computational challenges and adapting to emerging data types are crucial for the advancement of “ECEP.” By focusing on these areas, future research can enhance the model’s efficiency and predictive power, ultimately contributing to more precise and reliable enzymological studies.

## Discussion

Predicting enzyme turnover numbers is challenging due to small and noisy datasets. Bar-Even et al. (2011) discovered discrepancies of up to 20% between BRENDA entries and reference papers, possibly due to copying errors and unit replacements. Additionally, differences in *k*_cat_ measurements for identical enzyme reaction pairs in different studies can be significant, highlighting the challenge of ensuring consistency and accuracy in enzyme data. Our “ECEP” has performed remarkably well outperforming previous implementations, but it comes with its own set of limitations. The utilization of deep learning models demands significant computing power for optimizing hyperparameters and processing inferences, thereby impacting computational resources and time.

However, the presented results for “ECEP” signify more than just numerical values; they underscore the model’s capabilities. An *R*^2^ value of 0.55 indicates high predictive accuracy, suggesting adept capture of underlying enzyme-feature relationships, while a low MSE reinforces precision and alignment with observed values. These metrics, along with case studies, portray a robust, reliable, and nuanced model tailored to enzymology intricacies. In the broader enzymatic turnover prediction landscape, “ECEP” stands out not only due to superior metrics but also its unique approach. Compared to TurNuP and DLKcat, “ECEP” consistently outperforms, especially in complex enzyme families, sparse data, or non-linear dynamics scenarios. However, acknowledging the contributions of TurNuP and DLKcat is essential; they have laid the foundation for advancements in the field.

To further enhance “ECEP,” leveraging convolutional neural networks (CNNs) with powerful computing resources for hyperparameter optimization, acquiring more datasets, and refining feature engineering and extraction could be beneficial. Deep learning models thrive on ample data and sophisticated feature representation techniques, which can bolster predictive accuracy significantly.

In conclusion, the fusion of XGBoost and CNN Regression in the “ECEP” model presents a promising avenue for enzymatic turnover prediction. Despite challenges, its advantages and innovative approach position it as a transformative force in enzymology research. Its true impact will be realized through real-world applications and its contribution to advancing our comprehension of enzymes and their intricate dynamics.

## Conclusion

The journey of “ECEP” from conception to validation has been both enlightening and transformative. As we reflect on its performance, implications, and the road ahead, it’s evident that this model represents a significant leap in the realm of enzymatic turnover prediction.

- The introduction of “ECEP” has the potential to redefine the benchmarks in enzymatic turnover prediction. Its ability to provide nuanced, probabilistic outputs can guide experimental designs, risk assessments, and decision-making processes in enzyme kinetics research.
- The insights derived from the model, especially regarding feature importance, can pave the way for novel hypotheses, experimental designs, and a deeper understanding of enzyme dynamics.
- The model’s adaptability ensures that it remains relevant even as new data emerges, making it a valuable tool for ongoing and future research in the field.

Building on the foundations laid by models like TurNuP and DLKcat, future iterations of “ECEP” can explore hybrid approaches, amalgamating the strengths of different models to achieve even higher predictive accuracy.

In essence, “ECEP” represents a beacon of progress in the field of enzymatic turnover prediction. Its introduction promises not just enhanced predictive capabilities but also a deeper understanding of the intricate dance of enzymes. As with any scientific endeavor, the journey is ongoing, and “ECEP” is poised to lead the way, illuminating the path for future research and discoveries in enzyme kinetics.

In conclusion, our proposed methodology represents a significant advancement in the field of enzyme catalytic efficiency prediction. By shifting from simple mean averaging to a weighted mean approach, we address a critical limitation of previous models like TurNuP. Our weighted ensemble technique optimizes the contribution of each model based on its accuracy, resulting in more reliable and precise predictions. This innovative approach not only demonstrates superior performance but also offers a scalable and adaptable framework for future research in enzymology. Our findings underscore the importance of methodological improvements in predictive modeling, paving the way for more accurate and effective tools in bioinformatics.

## Data Availability

We used the BRENDA, UniProt, and Sabio-RK databases to create the *k*_cat_ dataset. All data and the code used to generate all results is publicly available only at https://github.com/misharisaud/ECEP. All figures are available in high quality at https://figshare.com/articles/figure/ecep-figures/27165615.
